# Detection of Host-Derived Sphingosine by *Pseudomonas aeruginosa* Is Important for Survival in the Murine Lung

**DOI:** 10.1371/journal.ppat.1003889

**Published:** 2014-01-23

**Authors:** Annette E. LaBauve, Matthew J. Wargo

**Affiliations:** Department of Microbiology and Molecular Genetics and The Vermont Lung Center, University of Vermont College of Medicine, Burlington, Vermont, United States of America; University of Washington, United States of America

## Abstract

*Pseudomonas aeruginosa* is a common environmental bacterium that is also a significant opportunistic pathogen, particularly of the human lung. We must understand how *P. aeruginosa* responds to the lung environment in order to identify the regulatory changes that bacteria use to establish and maintain infections. The *P. aeruginosa* response to pulmonary surfactant was used as a model to identify transcripts likely induced during lung infection. The most highly induced transcript in pulmonary surfactant, *PA5325* (*sphA*), is regulated by an AraC-family transcription factor, PA5324 (SphR). We found that *sphA* was specifically induced by sphingosine in an SphR-dependent manner, and also via metabolism of sphingomyelin, ceramide, or sphingoshine-1-phosphate to sphingosine. These sphingolipids not only play a structural role in lipid membranes, but some are also intracellular and intercellular signaling molecules important in normal eukaryotic cell functions as well as orchestrating immune responses. The members of the SphR transcriptome were identified by microarray analyses, and DNA binding assays showed specific interaction of these promoters with SphR, which enabled us to determine the consensus SphR binding site. SphR binding to DNA was modified by sphingosine and we used labeled sphingosine to demonstrate direct binding of sphingosine by SphR. Deletion of *sphR* resulted in reduced bacterial survival during mouse lung infection. In vitro experiments show that deletion of *sphR* increases sensitivity to the antimicrobial effects of sphingosine which could, in part, explain the in vivo phenotype. This is the first identification of a sphingosine-responsive transcription factor in bacteria. We predict that SphR transcriptional regulation may be important in response to many sites of infection in eukaryotes and the presence of homologous transcription factors in other pathogens suggests that sphingosine detection is not limited to *P. aeruginosa*.

## Author Summary

Many opportunistic pathogens transition from an environmental niche into the host. To establish an infection, these bacteria must rapidly adapt their transcriptional profile to the conditions at the site of infection. We used the response of *Pseudomonas aeruginosa* to lung surfactant as a model to discover genes important for bacterial survival during mouse lung infection. Using this model we identified transcripts induced in response to host-derived sphingolipids, accomplished by detection of the core component sphingosine by a sphingosine-binding transcription factor, SphR. Deletion of this transcription factor in *P. aeruginosa* reduced bacterial survival, highlighting the importance of a proper response to host-derived sphingosine. We present evidence that impaired survival against the antimicrobial effects of sphingosine may explain part of the in vivo survival defect of mutants in this response system. This is the first description of a specific bacterial response to sphingosine and its precursors, some of which are important immune signaling molecules. Thus, *P. aeruginosa* is capable of intercepting and responding to host immune modulatory signals. The importance of this response during infection and the presence of similar systems in other pathogens opens up a new avenue for investigation and expands our understanding of bacterial metabolic interactions with the host.

## Introduction

*Pseudomonas aeruginosa* is a common, Gram negative, environmental bacterium that can cause significant disease as an opportunistic pathogen, particularly in the lung. *P. aeruginosa* lung infections are prevalent in people with cystic fibrosis (CF) and chronic obstructive pulmonary disease (COPD), as well as individuals undergoing mechanical ventilation [1]–[4]. These infections cause significant morbidity and mortality and continue to be a major health care burden [5]–[8]. *P. aeruginosa* has a large genome by bacterial standards (∼6 Mbp) containing a high proportion of regulatory genes (∼8% are predicted to be transcriptional regulators) [9]. This large regulatory capacity is likely important for *P. aeruginosa* success as an opportunist, enabling it to rapidly alter gene expression in response to host-derived factors and environmental conditions. Understanding mechanisms by which *P. aeruginosa* detects and responds to the host could present new avenues to combat these devastating, and often antibiotic resistant [10], opportunistic infections. Our current understanding of *P. aeruginosa* response to the host come from transcriptional profiling using epithelial cells, mucus, or CF sputum [11], [12]. We were interested in the response of *P. aeruginosa* to the environment of the distal airway, particularly the response to mammalian pulmonary surfactant, the lipid rich mixture that coats the airway surface liquid of the lungs and participates in both respiratory physiology and host defense (reviewed in [13], [14]). This mixture is rich in phosphatidylcholine (∼75% by mass) but also has a substantial fraction of other phospholipids, cholesterol and its esters, and sphingolipids [15].

Sphingolipids constitute a class of molecules that are critical components of eukaryotic cell membranes. In addition to this structural role in membranes and their biophysical role in pulmonary surfactant, many sphingolipids have been shown to act as signaling molecules that play critical roles in regulation of diverse physiological processes. The broad importance of sphingolipid signaling in eukaryotic hosts has only recently been appreciated, and the rapidly expanding field has many recent reviews [16]–[19]. Sphingosine serves as a backbone component for all sphingolipids, which include the signaling molecules sphingosine-1-phosphate (S1P) and ceramide, as well as the structural lipid sphingomyelin. S1P, in particular, has been intensely studied in the past decade as a potent immune signaling molecule that plays a critical role in diverse immune functions such as lymphocyte trafficking, myeloid cell activation, and epithelial and endothelial barrier function, mediated by five G-protein coupled receptors [20]–[24]. Importantly, S1P is released by endothelial cells and platelets during the acute phase response and therefore plays an important role in the initial response to infection [25]–[28].

A specific transcriptional response to host derived sphingolipids and S1P has never been previously shown in bacteria. Here we have identified *P. aeruginosa* genes induced in response to mammalian pulmonary surfactant and subsequently characterized a subset of genes that are specifically and directly regulated by sphingosine or via metabolism of S1P, sphingomyelin, or ceramide to sphingosine. This response to sphingosine and its precursors is dependent on an AraC-family transcription factor in response to physiological levels of sphingosine and its precursors. This transcription factor binds sphingosine, which alters its association with DNA. A bacterial system to detect and respond to sphingosine may have broad implications in the modulation of host immune function and aid *P. aeruginosa* in altering host immune response in the human lung. In support of this prediction, deletion of the sphingosine-responsive transcription factor confers a survival defect during mouse lung infections.

## Results

### Transcriptional profiling of *P. aeruginosa* exposed to pulmonary surfactant (Survanta)

Microarray studies were used to identify a group of *P. aeruginosa* transcripts that were induced when the bacteria were grown in minimal media supplemented with lung surfactant (Survanta). When wild type PAO1 was exposed to minimal media containing lung surfactant compared to minimal medium with pyruvate alone, 125 transcripts (both predicted open reading frames (ORFs) and intergenic regions) were changed more than 3-fold (p<0.05), with 96 being induced (Table S1) and 29 being reduced (Table S2). Of the induced transcripts, 56 were characterized and 40 were predicted or hypothetical, while in the reduced transcript group, 11 were characterized and 18 were predicted or hypothetical. The induced class was dominated by genes from the Anr-regulon (29 genes, 16 of which were recently demonstrated as induced in surfactant [29]) and the choline catabolic pathway (15 genes) [30]–[32]. One observation of note in the induced group is the preponderance of transcripts encoding stress-related proteins including the chaperones *hslU*, *groEL*, *dnaK*, and *dnaJ*, and the universal stress protein family members *sspK*, *PA1789*, *PA4352*, and *PA5027*. We were interested in using the response to lung surfactant to identify gene function and novel biology in *P. aeruginosa*, and thus we have focused on highly induced genes of unknown function.

### *PA5325* induction by pulmonary surfactant and eukaryotic cells is in response to sphingosine

**Figure 1:**
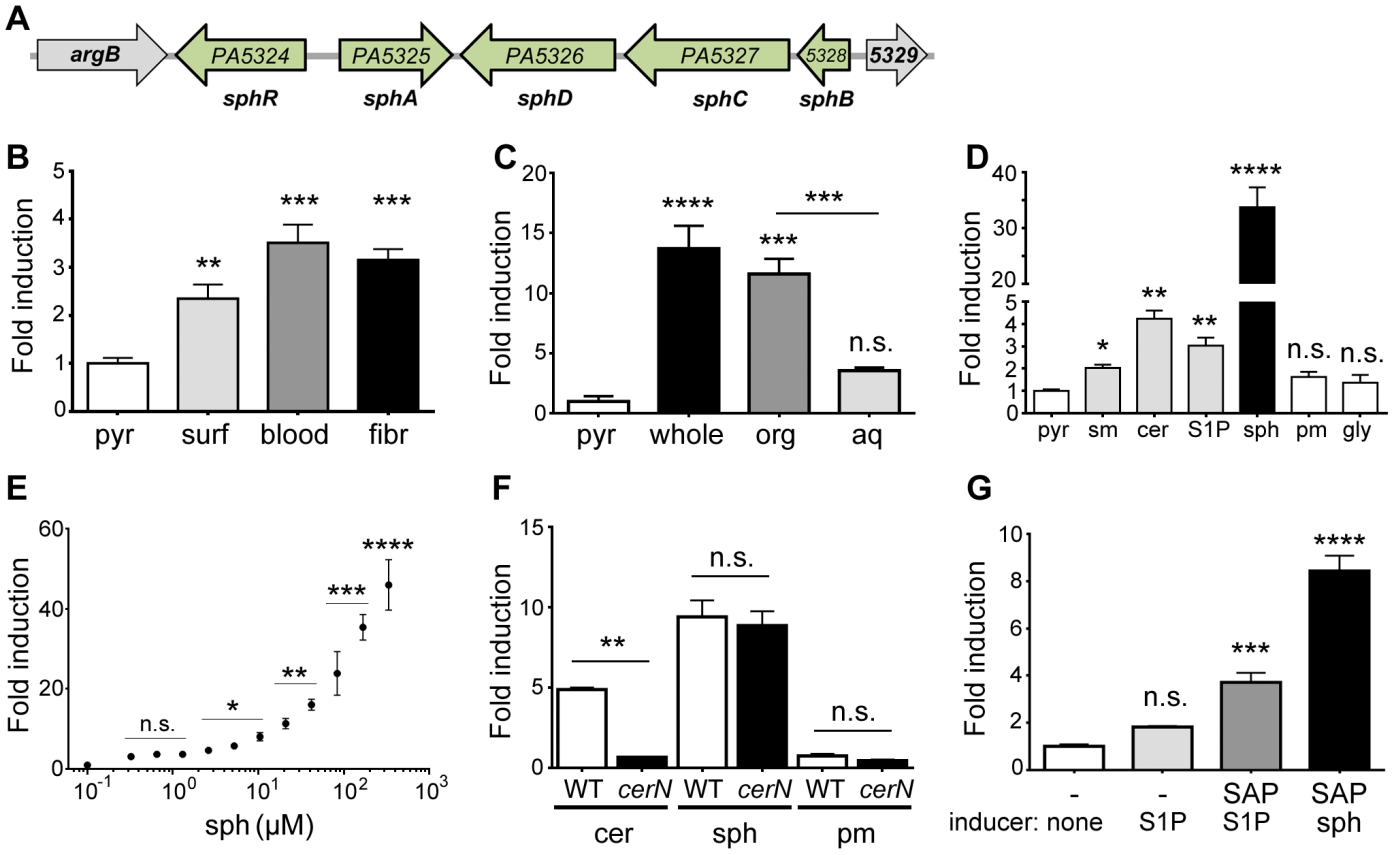
Expression of *sphA* (*PA5325*) is induced by sphingosine. (A) Arrangement of the *sphA* genomic region in *P. aeruginosa* PAO1. The genes in green are those discussed further in this study. Data from panels B through F all use a plasmid-borne *sphA*-*lacZ* reporter (pAL5) to assess regulation with pyruvate (pyr) used as the non-inducing control condition. Fold induction of *sphA-lacZ* is calculated compared to its pyruvate control. (B) *sphA* is induced in the presence of pulmonary surfactant (surf), sheep red blood cells (blood), and mouse fibroblasts (fibr). (C) The primary *sphA*-inducing component of fibroblasts is present in the organic fraction (org), compared to the aqueous fraction (aq) after the mouse fibroblasts (whole) were extracted with chloroform:methanol. (D) The sphingolipids sphingomyelin (sm), ceramide (cer), sphingosine-1-phosphate (S1P), and sphingosine (sph) induce *sphA*, while likely catabolic products palmitate (pm) and glycine (gly) do not cause induction. Sphingosine causes the highest level of induction. (E) Induction of *sphA* by sphingosine is dose dependent and occurs at physiologically relevant concentrations of sphingosine. Lines for statistical significance denote groups of samples with the same magnitude of significance, not grouped comparisons. (F) Induction of *sphA* by ceramide requires the neutral ceramidase gene, *cerN*, while sphingosine induction is independent of *cerN*. (G) Induction of *sphA* by S1P in a heterologous *E. coli sphR*-*sphA*-*lacZ* reporter system (pAL5) requires phosphatase treatment of S1P. Statistical significance determined using one way ANOVA with Dunnett's post-test for B–E & G with the uninduced condition being the comparator for all other data. In panel F, the wild type and Δ*cerN* data were compared by student t-test within each treatment condition. p-value summaries: n.s. = not significant; * for p<0.05; ** for p<0.01; *** for p<0.001; **** for p<0.0001. All experiments were performed at least three times and data shown is representative of both the scale and statistical significance levels of all experiments.

The *PA5325* transcript was induced ∼18 fold in the presence of lung surfactant and was the most highly induced transcript in these experiments (Table S1). The *PA5325* gene is divergently transcribed from *PA5324*, which encodes a probable AraC-family transcription factor that we hypothesized could be the transcriptional regulator of *PA5325* (Fig. 1A). The robust induction of *PA5325* in the presence of lung surfactant (Fig. 1B) suggested a possible role of this gene in the early stages of lung infection by *P. aeruginosa*.

For the following studies, we generated two reporter plasmids; pAL5 contained both *sphR* and the *PA5325*-*lacZYA* reporter and pAL4 contained only the *PA5325*-*lacZYA* reporter. Unless specified, the reporter used was pAL5 as it resulted in more robust induction. In addition to verifying the microarray results with surfactant, the *PA5325*-*lacZ* reporter was also induced in response to mouse fibroblasts (L-cells) and defibrinated sheep's blood (Fig. 1B). To determine which component of these eukaryotic-derived mixtures was a specific inducer of *PA5325*, we extracted mouse fibroblasts into aqueous and organic fractions and tested induction of *PA5325-lacZ*. The organic fraction contained the inducing activity, suggesting a lipid or other hydrophobic compound (Fig. 1C). Mouse fibroblasts and sheep's blood both contain high percentages of sphingomyelin [33], [34], and sphingomyelin makes up ∼4% of lung surfactant. Therefore, we tested induction of *PA5325* by sphingomyelin and related sphingolipids including ceramide, S1P, and sphingosine. *PA5325* was induced by sphingomyelin, ceramide, S1P, and sphingosine, but not the likely degradation products of sphingosine: palmitate and glycine (Fig. 1D). Other common lipid components of surfactant such as phosphatidylcholine and cholesterol did not induce transcription of *PA5325-lacZ*, and neither did unsaturated fatty acids (Supplemental Fig. S1). The strong induction by sphingosine compared to the other sphingolipids led us to hypothesize that the specific inducer of *PA5325* is sphingosine. We tested the sensitivity of our *PA5325*-*lacZ* reporter to sphingosine (Fig. 1E), which demonstrated *PA5325*-*lacZ* induction in a dose-dependent manner and showed response to physiological levels of sphingosine, which range from 200 nM (as S1P) in the serum and lymph up to 2–13 mM of free sphingosine in the skin and some epithelial surfaces [35], [36]. We did not reach saturation in this assay due to a combination of sphingosine insolubility, plastic binding, and bactericidal effects (discussed below).

We hypothesized that the reduced induction of *PA5325-lacZ* in response to sphingomyelin, S1P, and ceramide compared to sphingosine was due to a processing step required by *P. aeruginosa* to yield sphingosine. To test this hypothesis we generated a clean deletion in the neutral ceramidase encoded by *PA0845* and measured enzyme activity from the *PA5325-lacZ* reporter construct pAL4. Ceramide fails to induce *PA5325*-*lacZ* in the absence of the neutral ceramidase, whereas the response to sphingosine was unaffected (Fig. 1F). This finding strongly supports our hypothesis that induction of *PA5325* occurs in response to sphingosine. In addition, *PA5325-lacZ* is induced in response to S1P in *P. aeruginosa* (Fig. 1D), but not significantly induced by S1P in *E. coli* (Fig. 1G), although the reporter in *E. coli* could still be induced in the presence of sphingosine (Fig. 1G). This suggested that *P. aeruginosa* may be processing S1P and that *E. coli* does not possess an orthologous activity under these conditions. When S1P was pretreated with shrimp alkaline phosphatase, induction of *PA5325-lacZ* was partially restored in *E. coli* (Fig. 1G). Given the transcriptional control of *PA5325* in response to sphingosine, we have renamed it sphingosine regulated gene A, *sphA*.

### *sphA* transcription is dependent on *PA5324* (*sphR*)

**Figure 2:**
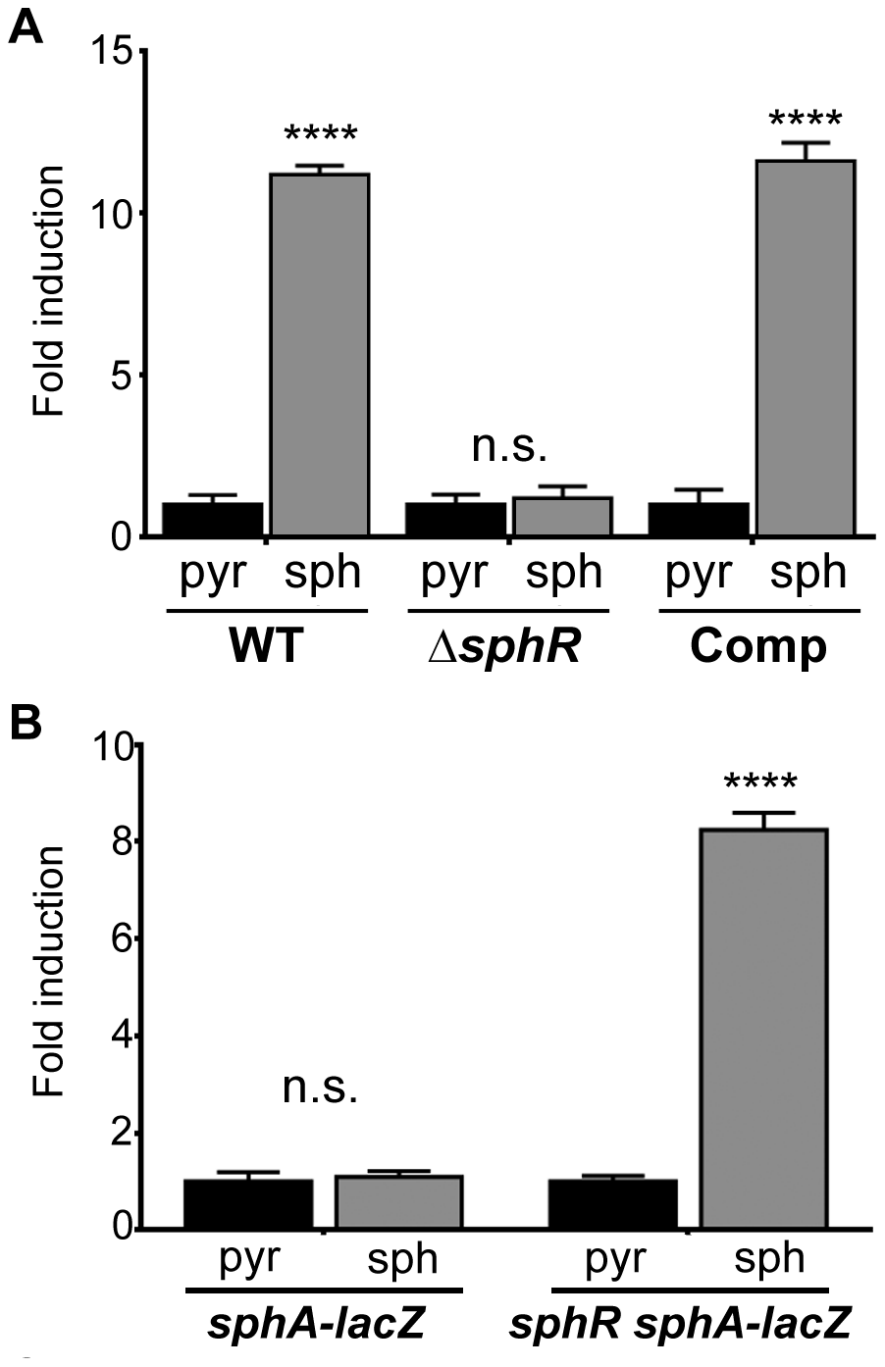
The transcription regulator SphR (PA5324) controls *sphA* induction in response to sphingosine. (A) Sphingosine (sph) induces *sphA*-*lacZ* expression (pAL4) compared to the pyruvate (pyr) control in wild-type cells (wild type) but not in *sphR* mutant cells (Δ*sphR*). This regulation is restored by complementation (Comp) of *sphR* at the *attTn7* locus. (B) In a heterologous *E. coli* system, the *sphA*-*lacZ* reporter (pAL4) is not responsive to sphingosine (second bar) unless the *sphR* gene is included in the system (pAL5) (fourth bar). Data for these panels were compared by student t-test comparing treatment conditions within each strain. p-value summaries: n.s. = not significant; **** for p<0.0001. All experiments were performed at least three times and data shown is representative of both the scale and statistical significance levels of all experiments.

*PA5324* encodes a predicted AraC-family transcription factor divergently transcribed from *sphA* (Fig. 1A). This arrangement led us to suspect that PA5324 was the transcriptional regulator of *sphA*. To confirm the requirement of *PA5324* for induction of *sphA* we generated an in-frame deletion of *PA5324*. The *PA5324* deletion strain carrying our *sphA*-*lacZ* reporter construct (pAL4) showed no induction in the presence of sphingosine (Fig. 2A). Insertion of *PA5324* onto the chromosome at the *attTn7* site restored induction in the deletion strain (Fig. 2A). Furthermore, *PA5324* was necessary to induce *sphA-lacZ* in a heterologous *E. coli* system in response to sphingosine (Fig. 2B). Our data suggest that the sphingosine responsiveness via *sphA* transcription is dependent on PA5324, and PA5324 was sufficient to confer sphingosine responsiveness in an *E. coli* system, therefore we have renamed PA5324 as the Sphingosine-responsive Regulator, SphR.

### Δ*sphR* mutants have a survival defect during mouse lung infection

**Figure 3:**
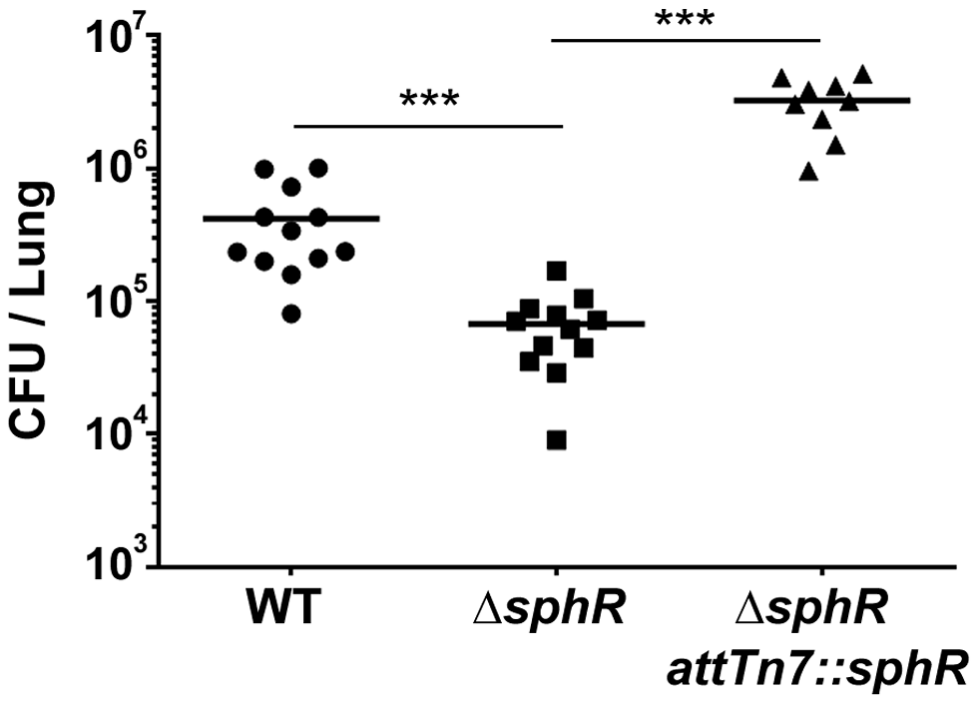
Deletion of *sphR* reduces *P. aeruginosa* survival in the mouse lung. Male C57Bl/6J mice were infected with 2×10^7^ CFU/mouse of each strain via oropharyngeal aspiration. Mice were euthanized and lungs harvested 24 hours after infection. Bacterial counts were determined by serial dilution onto Pseudomonas Isolation Agar (PIA). Deletion of *sphR* (Δ*sphR*) reduced *P. aeruginosa* survival 7.7-fold (p<0.001), an effect that was complemented by addition of *sphR* at the *attTn7* site. Wild-type (WT) and Δ*sphR* cells carried the empty *attTn7* insertion cassette as described in the methods section. Statistical significance determined using one way ANOVA with Tukey's post-test comparing all groups to each other. p-value summaries: n.s. = not significant; *** for p<0.001. Data shown is combined from two experiments. The same effect sizes and variance have been seen in at least one additional experiment for each strain, resulting in each strain having been compared to wild type in at least three independent experiments (described further in the Methods section).

Our *a priori* prediction was that SphR, controlling expression of a strongly induced gene by pulmonary surfactant, would be important for colonization and/or survival in the mammalian lung. To test this hypothesis, we examined bacterial survival 24 hours after infection in the mouse lung. The *sphR* deletion strain had significantly lower survival than wild type (7.7-fold decrease, Dunnett's multiple comparisons p<0.001) and the survival defect was complemented by addition of *sphR* at the *attTn7* site (Fig. 3). In this comparison, wild type and Δ*sphR* both contained the empty *attTn7* insertion cassette on the chromosome. The contribution of *sphR* to survival in the mouse lung led us to a more in-depth study of SphR and its target genes.

### Determination of the SphR regulon

Deletion of *sphR* resulted in reduced *P. aeruginosa* survival in the mouse lung (Fig. 3), leading us to hypothesize that one or more of the genes in the SphR regulon were likely candidates for this phenotype. To identify SphR-regulated genes in addition to *sphA*, we conducted microarray transcriptome analyses to compare wild type and the *sphR* deletion mutant in the presence and absence of pulmonary surfactant. Using a two-fold change cutoff and a p-value <0.05, there are six genes that differ between wild type and Δ*sphR* in the presence of surfactant (Table 1). Transcripts that are induced in wild type but not in the *sphR* deletion mutant include *sphA*, the neutral ceramidase (*PA0845*), and a three gene operon convergently transcribed toward *sphA*, *PA5328*-*PA5326*. The *argB* gene (*PA5323*) was induced more strongly in the *sphR* deletion than in wild type, which we think is likely due to a cis effect of the *sphR* (*PA5324*) deletion, as these genes are convergently transcribed (Fig. 1A). To denote their placement in the SphR regulon, we have renamed the genes in the predicted *PA5328*-*PA5326* operon as *sphBCD*. The *sphB* gene encodes a predicted periplasmic cytochrome and *sphC* and *sphD* encode a predicted flavin-dependent oxidoreductase and a predicted pyridoxalphosphate-containing threonine aldolase-like enzyme, respectively. The predicted functions of SphC and SphD suggest a potential two-step pathway for sphingosine degradation to glycine and a long chain aldehyde by oxidation to an aldol and subsequent cleavage by the aldolase, a prediction we are currently exploring. The neutral ceramidase (PA0845) was previously designated PaCD [37], which does not conform to standard bacterial nomenclature. We propose that *PA0845* be renamed *cerN* for ceramidase, neutral. The induction of *sphA* by surfactant in wild type (17.8-fold) versus the difference of *sphA* induction between wild type and Δ*sphR* (5.9-fold) suggested altered regulation of *sphA* in the absence of *sphR* (Table 1). The relative induction of *sphA* in the *sphR* mutant compared to wt under pyruvate (non-inducing) conditions supports a de-repression of *sphA* transcription in the absence of *sphR* at baseline. The remaining genes in the operon appear solely regulated by SphR under these conditions, as their induction levels in wild type compared to the difference between wild type and Δ*sphR* are not different.

**Table 1:** The SphR (PA5324) regulon in *P. aeruginosa.*

ORF/Feature	Gene name 1	Product Function	Fold Change WT Surf over sphR Surf	sphR Pyr over WT pyr	WT Surf over WT Pyr
*PA5327*	*sphC*	Predicted oxidase	10.6	NC	10.2
*PA5325*	*sphA*	Predicted porin	5.9	3.9	17.8
*PA5328*	*sphB*	Predicted cytochrome C (mono-heme)	5.0	NC	5.1
*PA0845*	*cerN*	Neutral ceramidase	3.1	NC	3.4
*PA5326*	*sphD*	Predicted threonine aldolase family	2.6	NC	2.9
*PA5323*	*argB*	Acetylglutamate kinase	−2.2	2.1	NC

^1^ gene names, apart from *argB*, all derived from this study.

### Mapping of the *sphA*, *sphBCD*, and *cerN* promoters

**Figure 4:**
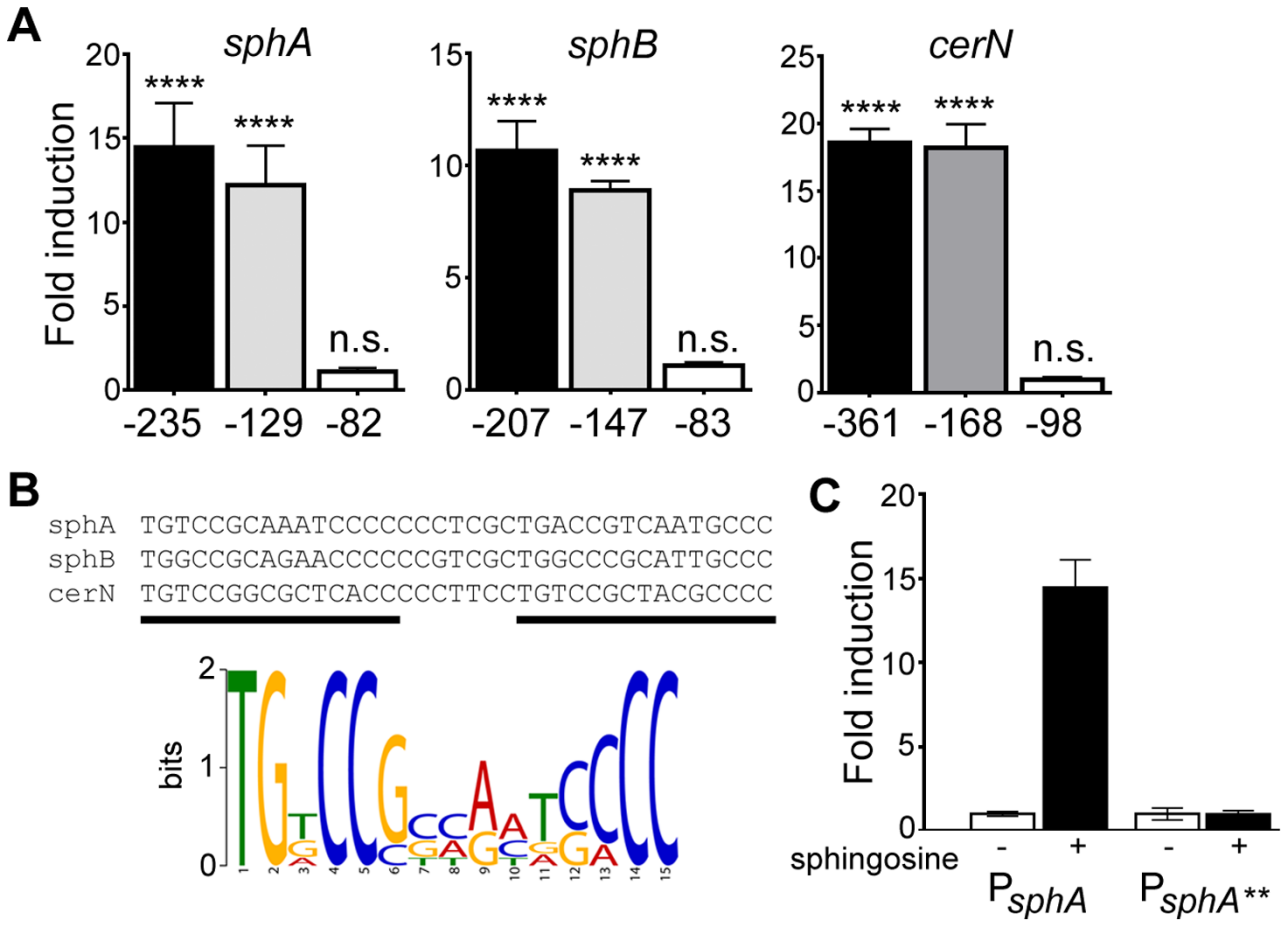
Determination of the probable SphR binding site from SphR-regulated promoters. (A) Fold-induction of ß-galactosidase activity from each reporter and the truncations compared to a pyruvate non-induced control (not shown). Promoter deletion mapping demonstrated a minimal region required for each SphR-controlled transcript. The negative numbers below each panel refer to the position relative to the translational start of each gene. (B) KALIGN showing the nucleotide alignment of the conserved region from the minimal regulatory regions defined in (A). The black bars denote the two direct repeat half-sites typical of AraC-family transcription factors. Below the alignments is the MEME-generated logo showing the strength of conservation based on the six half-sites from these three promoters. (C) ß-galactosidase assay for wt *sphA*-*lacZ* and the *sphA*-lacZ** promoter mutant (TG at the beginning of the first half-site was changed to AA) to demonstrate the importance of the conserved region for induction. Statistical significance determined using one way ANOVA with Dunnett's post-test with the uninduced pyruvate condition being the comparator for all other data. p-value summaries: n.s. = not significant; **** for p<0.0001. All experiments were performed at least three times and data shown is representative of both the scale and statistical significance levels of all experiments.

We used promoter mapping to identify the promoter proximal regions of the *sphA*, *sphBCD*, and *cerN* promoters that were important for sphingosine and *sphR*-dependent regulation. Using *lacZ* reporter fusions to each upstream region, we identified a portion of each promoter-proximal region required for responsiveness to sphingosine (Fig. 4A). The regions required for sphingosine responsiveness were aligned using KALIGN [38], which produced an alignment that highlights the general format of an AraC-family binding site (Fig. 4B). The MEME consensus for a single half-site is shown below the alignment (Fig. 4B). Bioinformatic search of the *P. aeruginosa* genome (DNA Motif Search [39]) turned up only one additional predicted binding site (two direct repeats of the consensus (TGNCCSNNRNNSNCC) separated by 6–8 bp) in the genome apart from those present in the three identified promoters. The additional binding site is in the intergenic region between *PA0428* and *PA0429*, upstream of the *PA0428* gene. We did not detect any change in the *PA0428* transcript for wild type or Δ*sphR* in the presence of surfactant or in either strain in the absence of surfactant. Therefore, based on our microarray data and bioinformatic analysis, we predict that *sphA*, *sphBCD*, and *cerN* likely comprise the core SphR regulon. The upstream sequences for the SphR regulon members showing the predicted SphR binding sites, promoter elements, and ribosome binding sites are shown in Supplemental Figure S2.

To test both specificity and the importance of conserved consensus sequences we mutated the first two residues in the consensus sequence TG to AA in half-site 1 (sphA**) (Fig. 4B), and tested the ability of the mutant sequence to permit induction of the reporter gene in response to sphingosine. The mutant reporter was unable to support reporter induction in response to sphingosine (Fig. 4C), demonstrating the importance of these conserved binding site residues.

### SphR directly binds the *sphA*, *sphBCD*, and *cerN* promoter proximal regions

**Figure 5:**
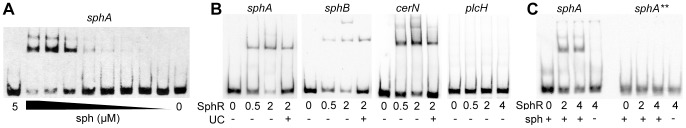
SphR directly binds to its target regulatory regions and binding is stimulated by sphingosine. The binding of MBP-SphR to target DNA was measured using electrophoretic mobility shift assays (EMSAs). (A) Sphingosine (sph) is required for robust MBP-SphR binding to target DNA and sphingosine stimulates SphR DNA binding in a dose-dependent manner. Based on this data all subsequent EMSAs were conducted with 10 µM sphingosine. (B) MBP-SphR (SphR) binds in a dose-dependent manner to *sphA*, *sphB*, and *cerN* upstream regulatory regions, but not to the *sphR*-independent *plcH* regulatory region. The MBP-SphR concentration (µM) is shown below the lanes. Specificity is shown by lack of MBP-SphR binding to the *plcH* promoter and by the ability to compete the bulk of the shift with unlabelled competitor DNA (UC) denoted by the + sign under the lanes. (C) MBP-SphR binds a 59-bp oligonucleotide probe containing the predicted SphR binding site upstream of *sphA*. The extra shifts seen in panels A & B are absent from the EMSAs with these minimal probes and likely are due to additional interaction sites. Mutation of two conserved residues in half-site #1 (TG to AA) (sphA**) results in substantial reduction in MBP-SphR binding. All experiments were performed at least three times and data shown is representative of all experiments.

We conducted electrophoretic mobility shift assays (EMSAs) with purified MBP-SphR fusion protein to test if SphR directly bound the *sphA*, *sphBCD, and cerN* promoters. The binding of MBP-SphR to the *sphA* promoter probe was greatly enhanced by the addition of sphingosine to the binding reaction in a concentration-dependent manner, providing evidence that sphingosine was a direct ligand of SphR (Fig. 5A). In the presence of sphingosine, MBP-SphR specifically shifted the *sphA*, *sphBCD*, and *cerN* promoters in a protein concentration-dependent manner and the binding could be competed with unlabeled *sphA* promoter probe, which gives a sense of the relative affinities for each binding site (Fig. 5B). MBP-SphR did not shift the non-specific *plcH* probe (Fig. 5B). The *plcH* probe is a useful negative control and demonstrates the specificity of SphR binding, as it has a known binding site for the AraC-family transcription factor GbdR in *P. aeruginosa* and its regulation is well described [31], [40]–[43].

To test the predicted SphR binding site, 59-mer oligonucleotides containing the proposed SphR binding site from the *sphA* promoter were annealed and the resultant probe was used in binding reactions. MBP-SphR was able to shift the 59-bp *sphA* probe (Fig. 5C, left), but only in the presence of sphingosine. Based on the inability of the mutated consensus sequence (sphA**) to support sphingosine-dependent reporter expression (Fig. 4C), we predicted that an oligonucleotide carrying these mutations would also be unable to bind SphR. As shown in the right side of Figure 5C, MBP-SphR was unable to bind this mutated probe. Together with the reporter fusions, these data support both the specificity of SphR binding and the importance of the conserved residues in the consensus.

### SphR binds sphingosine

**Figure 6:**
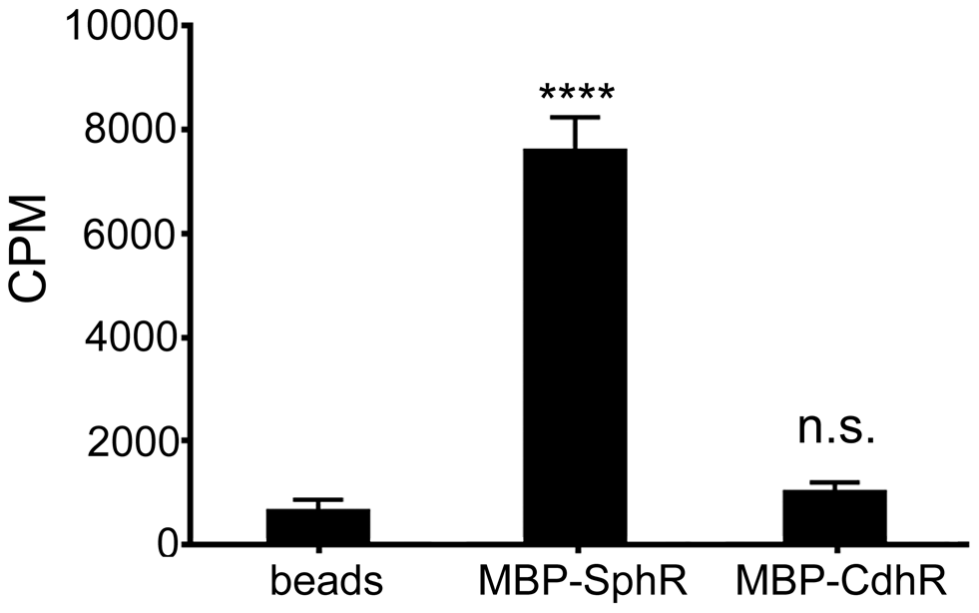
SphR directly binds to sphingosine. The association of MBP-SphR was determined by measuring binding of ^3^H-sphingosine and reporting counts per minute (CPM). Sphingosine minimally associates with amylose beads alone (beads) or an un-related AraC-family transcription factor (MBP-CdhR), but approximately 11-fold more ^3^H-sphingosine binds to MBP-SphR (p<0.0001). Statistical significance determined using one way ANOVA with Dunnett's post-test with the beads alone condition being the comparator for all other data. p-value summaries: n.s. = not significant; **** for p<0.0001. This experiment was performed at least three times and data shown is representative of both the scale and statistical significance levels of all experiments.

Based on the enhancement of SphR DNA binding in the presence of sphingosine and our genetic evidence, we predicted that SphR would directly bind sphingosine. We used ^3^H-sphingosine to test the ability of SphR to bind sphingosine (Fig. 6). The binding assay conditions were similar to those used for EMSA studies with MBP-SphR in the presence of ^3^H-sphingosine. Amylose resin beads were used to pull down the MBP-SphR, and bead-associated sphingosine was assayed by liquid scintillation counting. ^3^H-sphingosine was substantially enriched in the fraction containing amylose-bound MBP-SphR, while relatively little remained associated with the amylose beads alone, or beads bound to a non-specific MBP-tagged *P. aeruginosa* AraC-family transcription factor, CdhR (MBP-CdhR) [44]. These data, in combination with the EMSAs (Fig. 5), demonstrate direct interaction between sphingosine and SphR.

### Deletion of *sphA* phenocopies the mouse survival phenotype of the *sphR* deletion

**Figure 7:**
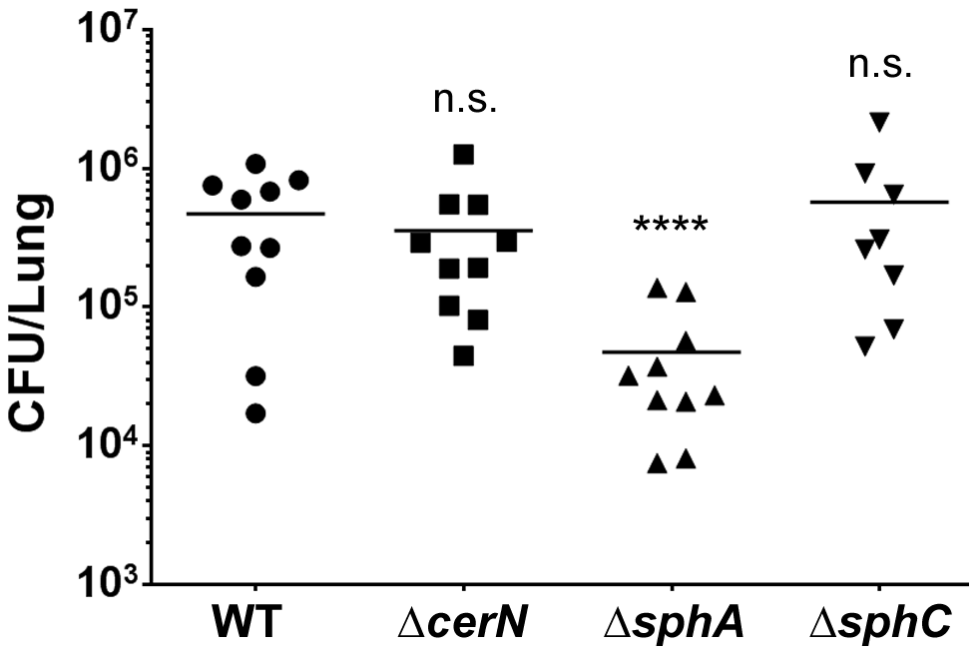
Deletion of the SphR-regulon member, *sphA*, reduces *P. aeruginosa* survival in the mouse lung. Male C57Bl/6J mice were infected with 2×10^7^ CFU/mouse of each strain via oropharyngeal aspiration. Mice were euthanized and lungs harvested 24 hours after infection. Bacterial counts were determined by serial dilution onto PIA. Deletion of *sphA* (Δ*sphA*) reduced *P. aeruginosa* survival 9-fold (p<0.001). Deletion of the other SphR-regulon members *cerN* and *sphC* (as an *sphBCD* operon representative) resulted in no survival defect. Statistical analysis determined using one way ANOVA with Dunnett's post-test with wild type as the comparator. Data shown is combined from two experiments. The same effect sizes and variance have been seen in at least one additional experiment for each strain, resulting in each strain having been compared to wild type in at least three independent experiments (described further in the Methods section).

**Figure 8:**
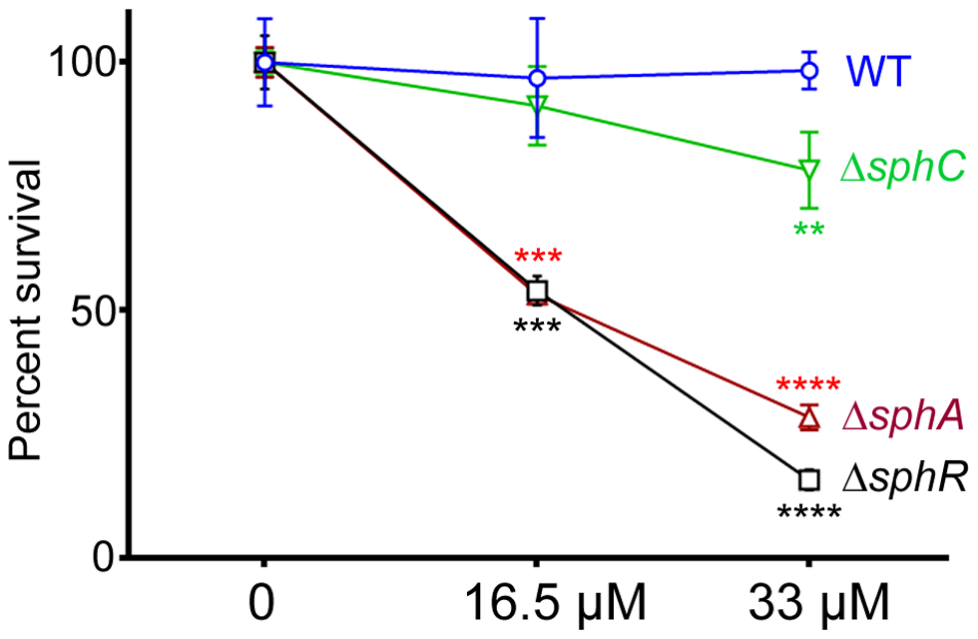
Deletion of *sphA* or *sphR* renders *P. aeruginosa* susceptible to killing by sphingosine. Strains with deletions in *sphA* (red), *sphR* (black), and *sphC* (green) were compared to wild type (blue) using a sphingosine killing assay in neopeptone as described in the Methods section. Cells were exposed to vehicle alone (0) or one of two concentrations of sphingosine for 30 minutes followed by serial dilution and plating. The mean of the vehicle treatment samples for each strain was set as 100% survival for that strain. Statistical significance determined using one way ANOVA with Dunnett's post-test with wild type at each concentration being the comparator for the mutant strain data at the same concentration. p-value summaries: n.s. = not significant; ** for p<0.01; *** for p<0.001; **** for p<0.0001. This experiment was performed more than three times and data shown is representative of both the scale and statistical significance levels of all experiments.

Because deletion of *sphR* led to reduced survival in the mouse lung, we were interested in determining which of the SphR regulon members contributed to survival in the lung. We generated deletions in *cerN*, *sphA*, and *sphC* and compared to wild type in our 24 hour lung infection model. Deletion of *sphA* led to a significant reduction in bacterial survival in the mouse lung (9-fold decrease, Dunnett's multiple comparisons p<0.001), while deletion of *cerN* or *sphC* had no impact on bacterial survival in vivo (Fig. 7). The sphA mutant phenotype could be complemented by supplying the *sphA* under its native promoter control at the *attTn7* site (Supplemental Fig. S3). These data suggest an important role for *sphA* in survival during infection. We did not test deletions of *sphB* and *sphD* in the animal model, given their predicted coordinate role with *sphC* in sphingosine metabolism and their similar phenotype to an *sphC* deletion during in vitro sphingosine killing (Fig. 8 and Figure S4).

### SphR & SphA are important for resistance to sphingosine killing

Sphingosine has previously been shown to have antimicrobial properties and is able to inhibit growth and kill many Gram positive and Gram negative bacteria [19]. Previous studies suggest that *P. aeruginosa* is not sensitive to killing by sphingosine [45]. We hypothesized that SphR might play a role in the response of *P. aeruginosa* to sphingosine and could regulate sphingosine resistance. Using a modified sphingosine killing assay, we show that the Δ*sphR* deletion strain is more sensitive to sphingosine compared to wild type (Fig. 8), an effect that could be complemented by *sphR* on a plasmid (Supplemental Fig. S4). Most of the sensitivity of the Δ*sphR* strain appears to be due to loss of *sphA* induction, as the Δ*sphA* strain is also more sensitive to sphingosine than wild type and is nearly as sensitive as Δ*sphR* (Fig. 8). The deletion phenotype of *sphA* could be complemented by *sphA* on a plasmid (Supplemental Fig. S4). Deletion of *sphC* and transposon insertions into *sphD* and *sphB* also led to small but reproducible decreases in survival on sphingosine, suggesting a minor role for this operon in the response to sphingosine (Fig. 8 and Supplemental Fig. S4). Deletion of *cerN*, befitting its known function as an extracellular ceramidase, had no effect on survival in sphingosine (data not shown).

## Discussion

The induction of ceramidase activity in response to sphingosine has been demonstrated in a few bacteria [37], but the mechanism of sphingosine detection and conversion into a response had not previously been elucidated. In this study we show that sphingosine is directly detected by the AraC-family transcription factor SphR (PA5324) leading to the induction of *sphA*, *sphBCD*, and *cerN* transcripts. Deletion of *sphR* or *sphA* resulted in survival defects in a mouse model of acute pneumonia, suggesting that the ability to detect and respond to host-derived sphingolipids is important for survival in the lung. Sphingolipids are abundant in mammals, plants, and fungi, constituting a diverse family of molecules that serve as essential structural components of eukaryotic cell membranes and as dynamic signaling molecules that mediate diverse cellular functions [16]–[19]. In particular, S1P has been implicated as a critical component of mammalian innate and adaptive immune function, particularly in the acute phase response to pathogens [25]–[28]. Interestingly, orthologs of SphR and some of the SphR-regulon members are present in other opportunistic pathogens including *Acinetobacter haemolyticus* and *Burkholderia pseudomallei*, as well as the professional pathogen *Mycobacterium tuberculosis*.

Sphingolipids play important roles in host-pathogen interactions, particularly S1P and ceramide signaling [46]–[48]. In addition to host modulation of sphingolipid pathways to combat infection, pathogens can modulate host sphingolipids. *M. tuberculosis* alters sphingolipid signaling in macrophages by undetermined mechanisms [49], and S1P levels in the lungs of patients infected with *M. tuberculosis* are significantly decreased [50]. Interestingly, *M. tuberculosis* has an AraC-family transcription factor that is 47% similar along the whole length to SphR (*RV1395*) that was identified though signature-tagged mutagenesis where the *RV1395* transposon mutant strain had an ∼1.5 log reduced survival in a mouse lung infection model [51]. Similarity between RV1395 and SphR is not restricted to the helix-turn-helix DNA-binding domain, as the two proteins are 44% similar when the DNA-binding domain is removed from the alignment analysis. RV1395 was characterized and found to be an activator of a divergently transcribed cytochrome gene, however the signals that govern RV1395 activation and its direct contribution to virulence have yet to be determined [52]. Based on the similarity of RV1395 to SphR we predict that a sphingolipid, perhaps sphingosine, may be the inducing ligand of RV1395.

The AraC-family transcription regulators are one of the largest groups of regulatory proteins in bacteria, and are often involved in the regulation of catabolism, stress response, and virulence [53]. Many members of the AraC family have been shown to respond to host-derived chemical signals present at the site of infection, but relatively few inducing ligands have been demonstrated to bind directly to their cognate regulator [54]. We found that addition of sphingosine altered the binding of SphR to the *sphA* promoter in EMSA studies and observed a dose response curve of SphR DNA binding at physiologically relevant concentrations of sphingosine. Bioinformatic analysis suggest similarity of SphR to ToxT (44% similarity and 20% identity), which directly regulates the major virulence factors in *Vibrio cholerae.* ToxT activation is inhibited by unsaturated fatty acids found in bile [55]. Subsequently, the crystal structure of ToxT was solved revealing a bound 16-carbon fatty acid that alters the structure of ToxT to prevent DNA binding in the presence of these bile associated fatty acids [56]. The similar size and hydrophobic nature of the regulatory ligands (palmitate vs. sphingosine) coupled with the sequence similarity allows us to speculate that SphR may bind sphingosine in a manner analogous to ToxT binding of palmitate.

Ito *et al.* identified a neutral ceramidase encoded by *PA0845* (renamed *cerN* in this study) that was induced in the presence of sphingomyelin, ceramide and sphingosine, however the regulatory mechanism was not reported [37]. The discovery of SphR control of neutral ceramidase allows us to expand a model of bacterial utilization of sphingomyelin by linking it to our previous work on regulation of the phospholipase C/sphingomyelinase PlcH. We previously characterized the AraC-family regulator GbdR that is integral to a positive feedback loop controlling PlcH expression in response to a metabolite of the choline headgroup of sphingomyelin [31]. Sphingomyelin hydrolysis by PlcH yields ceramide [57], which *P. aeruginosa* can further metabolize through the action of ceramidases [54]. Here we show that CerN is produced as part of an SphR-dependent positive feedback loop in response to the ceramide metabolite sphingosine, in a manner analogous to GbdR control of PlcH. Both of these positive feedback loops link induction of secreted catabolic enzymes not to the availability of the substrate itself, but to metabolic products derived from the substrate. In each case, this ensures that the positive feedback loop will robustly operate only if the substrate is being metabolized at sufficient rates.

Sphingolipids such as sphingosine have long been known to have antimicrobial properties and sphingosine is found in high concentration in the skin where it is thought to be part of the barrier function against microbial infections [58]–[61]. A variety of Gram positive and Gram negative bacteria are sensitive to sphingosine, including *Staphylococcus aureus* and *Escherichia coli* [62]. The precise bactericidal mechanism of sphingosine remains unknown. However, recent evidence suggests that sphingosine may directly damage bacterial membranes [63]. *P. aeruginosa* has recently been reported to be resistant to the bactericidal effects of sphingosine [45]. While none of the deletion strains generated in this study showed growth defects under normal conditions, we found that both the *sphR* and *sphA* deletion strains were susceptible to the antimicrobial effects of sphingosine compared to wild type in vitro. Strains with deletions in *sphR* and *sphA* were also shown to have reduced survival in the mouse lung. We hypothesize that the sensitivity of *sphA* and *sphR* mutants to sphingosine contributes to their observed reduced survival in vivo. It is interesting to note that the double deletion Δ*cerN*Δ*sphA* strain did not survive better or worse than Δ*sphA*, minimally suggesting that if the defect is due to sphingosine sensitivity, it is not sphingosine derived from *P. aeruginosa* hydrolysis of host-derived ceramide; in other words, they are not causing their own death by sphingosine derived from sphingomyelin and ceramide hydrolysis. Therefore, while the in vitro sphingosine killing correlates well with the in vivo phenotypes, we currently do not know the mechanism governing reduced survival of the *sphR* and *sphA* mutants in the lung.

We speculate that SphR responds to sphingosine to induce transcripts encoding proteins that protect *P. aeruginosa* from the bactericidal effects of sphingosine by induction of membrane stabilizing factors and/or catabolism of sphingosine to non-bactericidal metabolites. Here we show that SphR binds to sphingosine to initiate transcription of *sphA*, *sphBCD* and *cerN*. *sphA* encodes a hypothetical protein with some homology to proteins involved in meta-pathway phenol degradation. Protein localization predictions for SphA using the structure similarity-based prediction of Phrye2 [64] suggests that SphA is an outer membrane porin. Perhaps *P. aeruginosa* responds to sphingosine by providing a porin for sphingosine import and subsequent degradation that could aid in protecting the outer membrane from the damaging effects of free sphingosine. Okino and Ito demonstrated sphingosine utilization by *P. aeruginosa* by measuring removal of sphingosine from the culture supernatants and cell fractions [54]. Based on bioinformatic predictions, SphB, SphC and SphD are most likely involved in the metabolism of sphingosine. The *sphB* gene encodes a predicted cytochrome, while *sphC* encodes an FMN-linked oxidoreductase, and *sphD* encodes a pyridoxalphosphate serine-threonine aldolase. The latter two activities could work in concert to oxidize carbon 1, generating an aldol, which SphD could hypothetically act upon, rendering a long chain aldehyde and glycine. Transposon insertion into the *sphC* coding sequence (*PA5327*) resulted in reduced bacterial survival in a chronic rat lung infection model [65], suggesting that while our *sphC* deletion strain did not show a phenotype in the acute mouse lung infection (Fig. 7), it nonetheless impacts survival in the mammalian lung.

The microarray data comparing wild type in the presence and absence of pulmonary surfactant suggests some interesting biology in the presence of surfactant. The first observation has been covered by Jackson *et al*., who recently analyzed the changes in transcript levels of *P. aeruginosa* exposed to pulmonary surfactant, and compared wild type to both *plcH* and *gbdR* mutants [29], but did not publish results of these strains in the absence of pulmonary surfactant. They noted a reduction in transcript levels for Anr-controlled genes in both the *gbdR* and *plcH* mutants grown in surfactant, as do we (Table S1). Given the high levels of phosphatidylcholine and sphingomyelin in pulmonary surfactant, it was not surprising that the transcripts encoding proteins from the choline catabolic pathway were also highly induced in the presence of surfactant (Table S1). In addition to the high proportion of transcripts encoding stress-related proteins (mentioned in the Results section), there are also a high proportion (∼8%) of transcriptional regulators: NalC, BetI, NirG, PsrA, NarL, CgrA, PA3458, and PA4596. It is possible that the effects of induction of these transcription factors is contained in our regulation data, however our transcriptome analyses were a snapshot of transcripts at four hours post-induction and effects from changes in these transcription factors may not have sufficiently accumulated in the transcriptome. Of the reduced transcripts (Table S2), we note that three of the pyrroquinoline quinine biosynthesis genes are down, suggesting a change in requirement for this cofactor between surfactant and pyruvate conditions.

The demonstration of sphingosine detection by *P. aeruginosa* also opens up the possibility that this bacterium, and others with similar detection systems, could alter sphingosine and related sphingolipid signals, including S1P in the host. We have not yet examined the contribution of host immune signaling effected by the SphR regulon, but the impact of altering such an important and tightly controlled signaling network by bacterial factors has not been elucidated and may be an important contributing factor to the survival of *P. aeruginosa* in vivo.

## Materials and Methods

### Ethics statement

This study was performed in strict accordance with the recommendations in the Guide for the Care and Use of Laboratory Animals of the National Institutes of Health. The protocol for animal infection was approved by the University of Vermont Institutional Animal Care and Use Committee (Permit number A3301-01). All procedures were performed under pentobarbital anesthesia and all efforts were made to minimize animal suffering.

### Strains, growth conditions, and chemicals

*P. aeruginosa* PAO1, isogenic mutant strains, and *E. coli* (Table 2) were maintained in LB-Lennox (LB) medium. Morpholinepropanesulfonic acid (MOPS) medium [66] supplemented with 25 mM sodium pyruvate, 5 mM glucose and 50 µg/ml gentamicin (for *P. aeruginosa*) or MOPS with 10% LB (v/v), 5 mM glucose, and 10 µg/ml gentamicin (for *E. coli*) was used to grow strains prior to transcriptional induction studies. See LaBauve and Wargo (2012) for further details on *P. aeruginosa* growth methods [67]. For bactericidal assays, 1% neopeptone was supplemented with varying sphingosine concentrations in ethanol to reach a final concentration of 6.25% (w/v) ethanol in the assay. All lipids were purchased from Avanti Polar Lipids and other chemicals were purchased from Sigma-Aldrich or Fisher.

**Table 2:** Bacterial strains and plasmids used in this study.

Organism/Plasmid	Description/Genotype	Name	Source
*Pseudomonas aeruginosa*	PAO1 wild type	MJ79	[75]
	Δ*sphR*	AL76	This study
	Δ*sphA*	AL82	This study
	Δ*sphC*	AL119	This study
	Δ*cerN*	AL114	This study
	Wild type empty::*attTn7* Gm^R^	MJ507	This study
	Δ*sphR* empty::*attTn7* Gm^R^	MJ508	This study
	Δ*sphR sphR*::*attTn7* Gm^R^	MJ540	This study
	Wild type empty::*attTn7* Gm^S^	AL138	This study
	Δ*sphR* empty::*attTn7* Gm^S^	AL139	This study
	Δ*sphR sphR*::*attTn7* Gm^S^	AL140	This study
*Escherichia coli*	NEB5α		NEB
	S17-1 λ*pir*	MJ340	[76]
Plasmid	*sphA*-*lacZYA* reporter	pAL4	This study
	*sphR*-*sphA*-*lacZYA* reporter	pAL5	This study
	MBP-SphR expression vector	pAL11	This study
	Maltose binding protein (MBP) expression vector	pMAL-c2	NEB
	Integration vector	pMQ30	Shanks
	*P. aeruginosa* replicative *lacZYA* reporter	pMW5	Wargo
	*P. aeruginosa*-yeast shuttle *lacZYA* reporter	pMW42	This study
	*sphR*::*attTn7* integration vector	pMW118	This study
	Empty::*attTn7* integration vector	pUC18-mini-Tn*7*T-Gm	Schweizer

### Mouse lung infection model

We used the oropharyngeal route of mouse lung infection previously described [68], [69]. Briefly, *P. aeruginosa* PAO1 and isogenic strains were streaked onto LB plates from −80°C stocks. Colonies from the first plate were restreaked onto a new LB plate after 24 hours and incubated at 37°C for 24 hours. Cells from the second plate were used to start 3 ml cultures in LB that were grown for 16–18 hours at 37°C on a roller drum. From these overnight cultures, cells were collected by centrifugation, washed in Dulbecco's PBS (DPBS), and resuspended to give ∼1×10^7^ viable *P. aeruginosa* in 40 µL, with actual inoculum determined by serial dilution and plate counting. Eight to twelve week old male C57Bl/6J mice (Jackson Labs) were inoculated with 40 µL of the bacterial suspension via oropharyngeal aspiration. Anesthesia, surgery, bronchoalveolar lavage fluid (BALF) collection, organ harvest, and organ homogenization were done as previously described [68], [69] at 24 hours post-infection. Viable bacterial counts in organs were determined by serial dilution plating onto Pseudomonas Isolation Agar (PIA) (BD-Difco) followed by incubation at 37°C for 24 hours.

Mouse experiments (Fig. 3 and 7 and Supplemental Fig. S3) show CFU counts from all animals from duplicate experiments with each replicate having 4–6 animals per experimental group. All informative comparisons: mutants versus wild type (both Figures) and mutant versus complementation strain (Fig. 3 and Supplemental Fig. S3) were conducted in at least one additional experiment, included with comparator strains from other studies. Therefore, all informative comparisons were assessed three times. All experiments met the same statistical criteria, i.e. all replicates were consistent with regards to effect size and significance of changes. Inoculation order and harvest order alternated between experiments to eliminate potential issues related to the difference between the duration of inoculation (∼20–30 min) and the duration of harvest (∼1.5 h). For group comparisons, data (log_10_ transformed CFU counts) were analyzed by ANOVA followed by Tukey's (Fig. 3 and Supplemental Fig. S3) or Dunnett's (Fig. 7) Multiple Comparisons tests. All calculations were done using GraphPad Prism.

### RNA extraction and microarray methodology

*P. aeruginosa* PAO1 wild type and Δ*sphR* were grown overnight in MOPS media supplemented with 20 mM pyruvate and 5 mM glucose. Overnight cultures were collected by centrifugation and resuspended in either MOPS supplemented with 20 mM pyruvate alone or 20 mM pyruvate and a 1∶50 dilution of the bovine surfactant preparation Survanta (Abbott) and induced for 4 hours at 37°C. Bacteria were collected by centrifugation, resuspended in MOPS and RNA Protect Bacterial Reagent (Qiagen), and the resultant pellets stored overnight at −20°C. RNA was extracted using an RNeasy kit (Qiagen), and eluted samples were treated with DNase I followed by a second round of RNeasy purification including an on-column DNase I treatment. Purified RNA samples were checked for DNA contamination by PCR and RNA integrity scores based on Agilent Bioanalyzer analysis were indicative of little to no DNA contamination.

Microarray analysis was performed on a *Pseudomonas aeruginosa* PAO1 gene chip using raw oligonucleotide probes generated from each condition using the NuGen Pico system. Each condition was analyzed in duplicate (N = 2), and summarized in one probe intensity by the Vermont Genetics Network Microarray Facility using Affymetrix GCOS software. Information from multiple probes was combined to obtain a single measure of expression for each probe set and sample. Probe-level intensities were background-corrected, normalized, and summarized, and Robust Multichip Average (RMA) statistics were calculated for each probe set and sample as is implemented in Partek Genomic Suites, version 6.6 (Copyright 2009, Partek Inc., St. Louis, MO, USA). Sample quality was assessed based on relative log expression (RLE), and normalized unscaled standard error (NUSE). To identify differentially expressed genes, linear modeling of sample groups was performed using ANOVA as implemented in Partek Genomic Suites. The magnitude of the response (fold change calculated using the least square mean) and the p-value associated with each probe set and binary comparison were calculated. The data have been submitted to NCBI GEO with accession number GSE48982.

### Construction of deletion strains and complementation

Deletion mutants were generated using the pMQ30 plasmid [70] carrying the flanking regions of each of the four genes, *sphR*, *sphA*, *sphC*, and *cerN*, using conjugation-mediated deletion as described previously [30], [69]. Primers for these constructs are listed in Table S3. Single cross-over mutants were selected on PIA with gentamicin and selection of double crossover deletion mutants were carried out on LB 5% sucrose plates prepared without NaCl. Unmarked deletion mutants were verified using PCR. Complementation was done by integration of the *sphR* or *sphA* coding sequence under control of their native promoter at the *attTn7* locus using the pUC18-miniTn7T-Gm vector as we described previously [68], [69] using the method of Choi and Schweizer [71]. This allowed stable complementation in the absence of antibiotic. For complementation where reporter plasmids were used, the gentamicin resistance cassette was excised by FLP-mediated recombination [71]. All *sphR*::*attTn7* and *sphA::attTn7* complementation strains were compared with wild type or mutant strains carrying the empty *attTn7* integration region from the pUC18-miniTn7T-Gm vector.

### Reporter assay to measure *sphA* transcriptional induction

Two reporter constructs were generated in this study using yeast homologous recombination [70] to generate translational fusions to *lacZYA*. A target *lacZYA*-containing vector suitable for yeast cloning (pMW42) was generated by excising the *lacZYA* region from pMW5 [31] with HindIII and EcoRI and cloning into the similarly cut pMQ80 backbone [70], which removes *egfp*-*mut3*. Either the *sphA* promoter (pAL4), or the entire *sphR* gene and the *sphA* promoter (pAL5) were recombined with pMW42 linearized with KpnI and HindIII. *P. aeruginosa* strains were electrotransformed with the reporter constructs and grown overnight in MOPS media supplemented with 20 mM pyruvate, 5 mM glucose, and 50 µg/ml gentamicin prior to induction. Inductions were carried out in MOPS media supplemented with 20 mM pyruvate and the inducing compound and incubated at 37°C for 6 hours. β-galactosidase assays were done as previously described [31], [72], using the method of Miller [73]. Studies of heterologous *sphA* induction in *E. coli* were carried by transforming pAL4 and pAL5 into *E. coli* NEB5α. Resulting *E. coli* strains were grown overnight in MOPS media supplemented with 10% LB (v/v), 5 mM glucose and 10 µg/ml gentamicin. For induction assays with S1P in *E. coli*, 2.4 µg of S1P or sphingosine were pre-treated with or without 5 U shrimp alkaline phosphatase (SAP) in 100 µL of water with 1× SAP buffer (USB), and incubated at 37°C for 60 minutes. Induction assays were carried out in MOPS supplemented with 10% LB (v/v), treated inducing compounds, and 10 µg/ml gentamicin. All *E. coli* strains were induced for 8 hours prior to ß-galactosidase assays.

### Promoter mapping of *sphA*, *sphB*, and *cerN*

Full-length reporter constructs and truncations of *sphA*, *sphB*, and *cerN* promoters were cloned into pMW5 [31]. The resultant *lacZYA* reporter constructs were transformed into wild type *P. aeruginosa* and used to identify the region required for response to sphingosine. Inductions were carried out in MOPS media supplemented with 20 mM pyruvate and 150 µM sphingosine and incubated at 37°C for 6 hours followed by ß-galactosidase assays.

### Cloning, expression and purification of SphR

We constructed a maltose binding protein (MBP) fusion to SphR by using the pMALc2 vector system (NEB). The *sphR* gene was amplified from genomic DNA. The PCR product was gel purified and ligated into the pCR Blunt vector (Invitrogen). The insert was excised with KpnI and HindIII, gel purified, and ligated into a similarly digested pMALc2 vector to generate pAL11. *E. coli* NEB5α (New England Biolabs) carrying the pAL11 plasmid were grown overnight in LB supplemented with 120 µg/ml carbenicillin. The overnight culture was transferred to two 500 ml flasks containing 100 ml of LB-carbenicillin and shaken at 220 rpm for 5 hours. Isopropyl-β-D-thiogalactopyranoside (IPTG) was added to a final concentration of 1 mM, and the cells were induced for 3 hours. Cells were collected by centrifugation, lysed in column buffer (20 mM Tris-HCl, pH 7.5, 150 mM NaCl, 1 mM EDTA) supplemented with 3 mg/ml lysozyme and Halt protease inhibitor 1× cocktail (Thermo Scientific). Lysates were clarified by centrifugation, and the soluble fraction was applied to a column containing amylose resin (NEB). The column was washed with ten volumes of column wash buffer (20 mM Tris-HCl, 150 mM NaCl 1 mM EDTA pH 7.4), followed by elution with column wash buffer supplemented with 10 mM maltose. Elution fractions were run on 10% SDS-PAGE gels and visualized by Coomassie staining. Fractions containing the MBP-SphR were pooled and dialyzed against 20 mM Tris-HCl, pH 7.5 at 4°C in a 20,000 kDa cutoff Slide-A-lyzer cassette (Pierce). The full length MBP-SphR fusion protein was used in electrophoretic mobility shift assays, as the MBP tag did not prevent sequence specific DNA binding (Fig. 5) or binding to sphingosine (Fig. 6).

### Electrophoretic Mobility Shift Assay (EMSA)

EMSA DNA probes were generated using PCR (Primers in Table S3) and were spot dialyzed against 2.5 mM Tris-HCl, 0.25 mM EDTA, pH 8.0. Labeled probes, generated using a primer with a covalently linked 5′ biotin tag (IDT), were used at 0.5 fmol/µl, and unlabeled competitor probes were used at a final concentration of 0.5 pmol/µl. EMSA was carried out using a Thermo Scientific Thermoshift kit. The final binding buffer was modified to contain 1× binding buffer (10 mM Tris-HCl, pH 7.5, 50 mM KCl, 1 mM dithiothreitol), 0.1 mM glycine betaine, and 2 µg/ml poly-dI-dC. Various concentrations of sphingosine dissolved in ethanol were added to reaction tubes and allowed to dry to eliminate ethanol prior to binding reactions. Binding reactions were carried out at 37°C for 15 minutes and electrophoresed on a 5% non-denaturing polyacrylamide gel then transferred to a BioDyne B membrane (Thermo Scientific). Detection was carried out using streptavidin-linked horseradish peroxidase according to the supplied protocol (Thermo Scientific).

### Measurements of sphingosine association with SphR

Sphingosine association with SphR was measured by conducting binding reactions using ^3^H-D-erytho-sphingosine (Perkin-Elmer). Binding reactions were carried out as described for EMSA except ^3^H-D-erytho-sphingosine was used at a final concentration of 50 nM. Samples were incubated with and without either 10 µM MBP-SphR or 10 µM MBP-CdhR for 30 minutes then added to amylose resin. The amylose beads were collected by centrifugation and washed 3 times with amylose column wash buffer. After washes, amylose beads were resuspended in 200 µl of amylose wash buffer and transferred to a glass vial containing 10 ml of Biosafe II scintillation cocktail (RPI). Samples were quantified using a Tri-Carb 2910 TR liquid scintillation analyzer (Perkin-Elmer).

### Sphingosine killing assay

Killing assays were carried out as previously described [61]. Briefly, overnight *P. aeruginosa* strains were grown in trypticase soy broth (TSB) and diluted 1∶40. Diluted cultures (100 µl) were added to glass tubes containing 250 µl of 1% neopeptone supplemented with 50 µl of the appropriate sphingosine stock in ethanol or ethanol alone as the vehicle control. The cultures were shaken at 170 rpm for one hour. Survival was determined by serial dilution plating on PIA. Colonies were counted after 24 hour incubation and survival calculated by comparison to vehicle only controls.

## Supporting Information

Figure S1 **Induction of sphA-lacZ in response to inducing lipids of varying lengths, structures, and saturation.** Growth, induction, and ß-galactosidase assays conducted as described in the methods section using *P. aeruginosa* carrying the pAL5 reporter plasmid. All compounds were used at a final concentration of 150 µM with methanol as the vehicle for linoleic acid (final vehicle concentration 0.05%) and ethanol as the vehicle for all other compounds (final vehicle concentration 0.05%).
(TIF)

Figure S2 **DNA sequences for the 400-base pairs upstream of the** ***sphA*** **,** ***sphB*** **, and** ***cerN*** **genes.** The ATG at the end of each sequence marks the start-codon of the labeled gene. Underlined normal text indicated predicted ribosome binding sites. The teal-blue highlighted bases indicate predicted −10 promoter elements. The potential −35 elements were further from consensus in these promoters and are not indicated on this figure. The green highlighted sequences indicate the SphR binding site. For the *sphA* region, the *sphR* start codon is marked, as is the transcriptional start site (underlined bold italic) as determined by Wurtzel et al. [74]. The other transcriptional start sites were not detected in the Wurtzel study, likely due to the absence of a suitable inducer.
(TIF)

Figure S3 **Deletion of** ***sphA*** **reduces** ***P. aeruginosa*** **survival in the mouse lung.** Male C57Bl/6J mice were infected with 2×10^7^ CFU/mouse of each strain via oropharyngeal aspiration. Mice were euthanized and lungs harvested 24 hours after infection. Bacterial counts were determined by serial dilution onto Pseudomonas Isolation Agar (PIA). Deletion of *sphA* (Δ*sphA*) reduced *P. aeruginosa* survival ∼7-fold, an effect that was complemented by addition of *sphA* under control of its native promoter at the *attTn7* site. Wild-type (WT) and Δ*sphA* cells carried the empty *attTn7* insertion cassette (*attTn7-E*) as described in the methods section. Statistical significance determined using one way ANOVA with Tukey's post-test comparing all groups to each other. p-value summaries: * for p<0.05, ** for p<0.01. Data shown is combined from two experiments.
(TIF)

Figure S4 **The survival of SphR-regulon mutants in sphingosine.** Strains with deletions in *sphA* (A) and *sphR* (B) were susceptible to sphingosine killing and each deletion could be partially complemented with the respective gene on a plasmid compared to the empty vector (pEmpty). (C) Transposon mutants in *sphB*, *sphD*, and *sphR*, and a deletion mutant of *cerN* were compared to WT for sphingosine susceptibility. Statistical significance determined using one way ANOVA with Dunnett's post-test with wild type at each concentration being the comparator for the mutant strain data at the same concentration. p-value summaries: * for p<0.05; ** for p<0.01; *** for p<0.001; **** for p<0.0001. These experiments were performed more than three times and data shown is representative of both the scale and statistical significance levels of all experiments.
(TIF)

Table S1 ***P. aeruginosa*** **transcripts induced more than 3-fold in response to pulmonary surfactant.**
(XLSX)

Table S2 ***P. aeruginosa*** **transcripts reduced more than 3-fold in response to pulmonary surfactant.**
(XLSX)

Table S3 **Primers used in this study.**
(XLSX)
