# Supplementary figures and images for: Detection of Host-Derived Sphingosine by Pseudomonas aeruginosa Is Important for Survival in the Murine Lung
Source: PLoS Pathog. 2014 Jan 23;10(1):e1003889. doi: 10.1371/journal.ppat.1003889 (PMC3900636; doi:10.1371/journal.ppat.1003889)

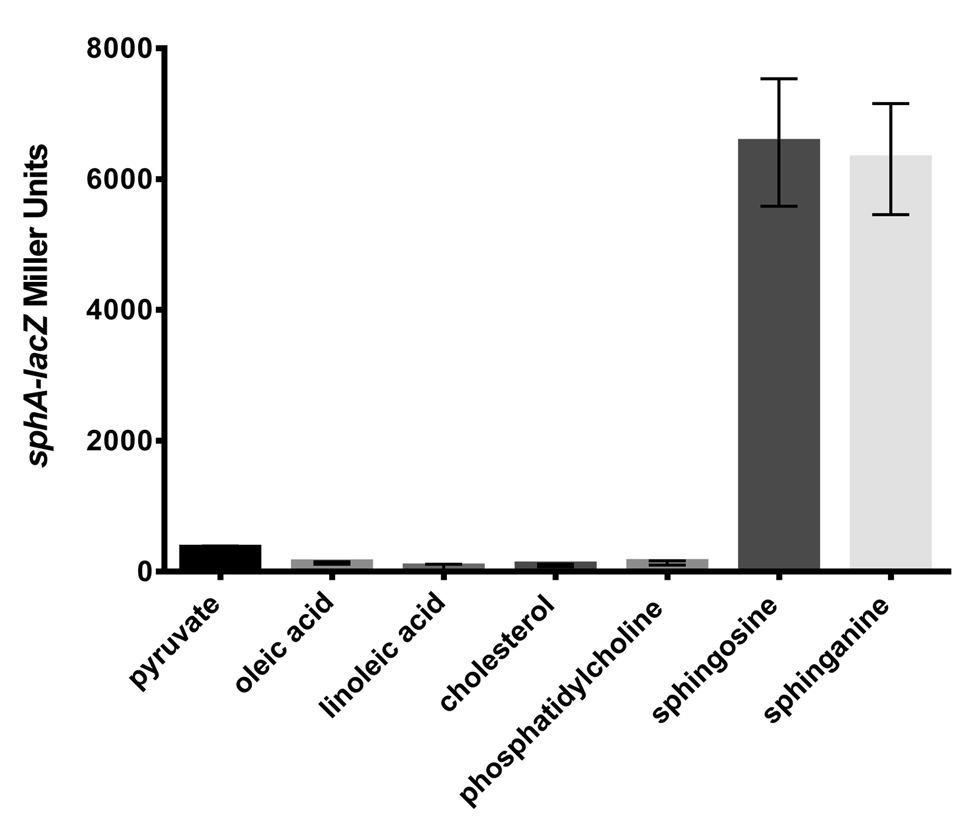

Supplement: Figure S1 — Induction of sphA-lacZ in response to inducing lipids of varying lengths, structures, and saturation. Growth, induction, and ß-galactosidase assays conducted as described in the methods section using P. aeruginosa carrying the pAL5 reporter plasmid. All compounds were used at a final concentration of 150 µM with methanol as the vehicle for linoleic acid (final vehicle concentration 0.05%) and ethanol as the vehicle for all other compounds (final vehicle concentration 0.05%). (TIF) [file ppat.1003889.s001.tif]

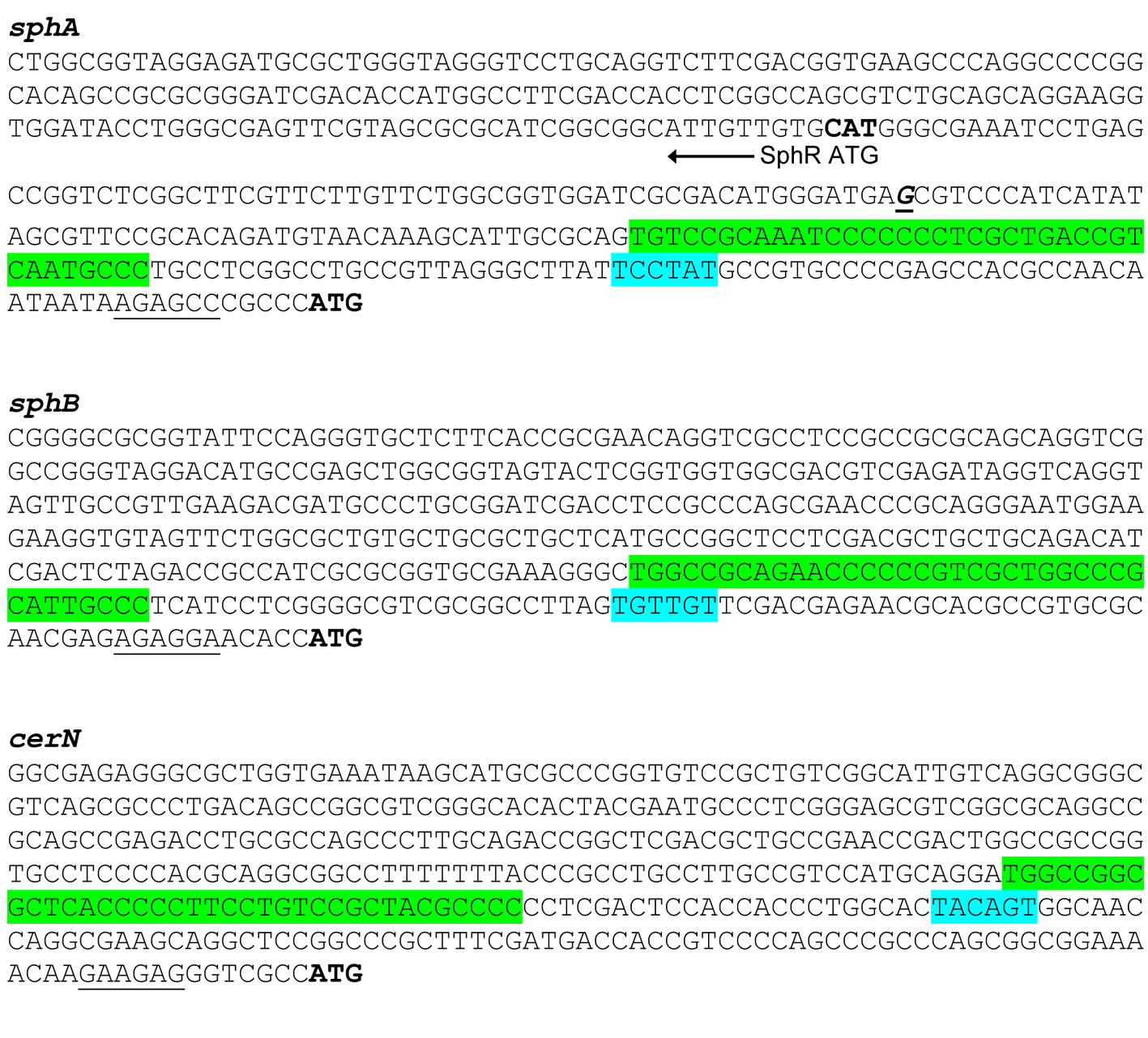

Supplement: Figure S2 — DNA sequences for the 400-base pairs upstream of the sphA , sphB , and cerN genes. The ATG at the end of each sequence marks the start-codon of the labeled gene. Underlined normal text indicated predicted ribosome binding sites. The teal-blue highlighted bases indicate predicted −10 promoter elements. The potential −35 elements were further from consensus in these promoters and are not indicated on this figure. The green highlighted sequences indicate the SphR binding site. For the sphA region, the sphR start codon is marked, as is the transcriptional start site (underlined bold italic) as determined by Wurtzel et al. [74]. The other transcriptional start sites were not detected in the Wurtzel study, likely due to the absence of a suitable inducer. (TIF) [file ppat.1003889.s002.tif]

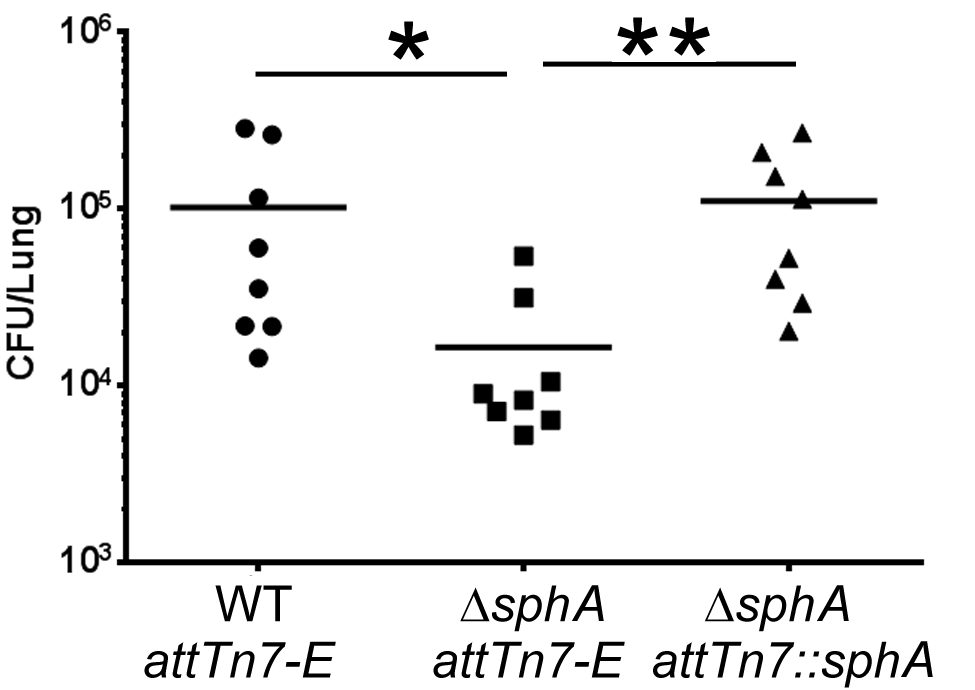

Supplement: Figure S3 — Deletion of sphA reduces P. aeruginosa survival in the mouse lung. Male C57Bl/6J mice were infected with 2×10^7^ CFU/mouse of each strain via oropharyngeal aspiration. Mice were euthanized and lungs harvested 24 hours after infection. Bacterial counts were determined by serial dilution onto Pseudomonas Isolation Agar (PIA). Deletion of sphA (ΔsphA) reduced P. aeruginosa survival ∼7-fold, an effect that was complemented by addition of sphA under control of its native promoter at the attTn7 site. Wild-type (WT) and ΔsphA cells carried the empty attTn7 insertion cassette (attTn7-E) as described in the methods section. Statistical significance determined using one way ANOVA with Tukey's post-test comparing all groups to each other. p-value summaries: * for p<0.05, ** for p<0.01. Data shown is combined from two experiments. (TIF) [file ppat.1003889.s003.tif]

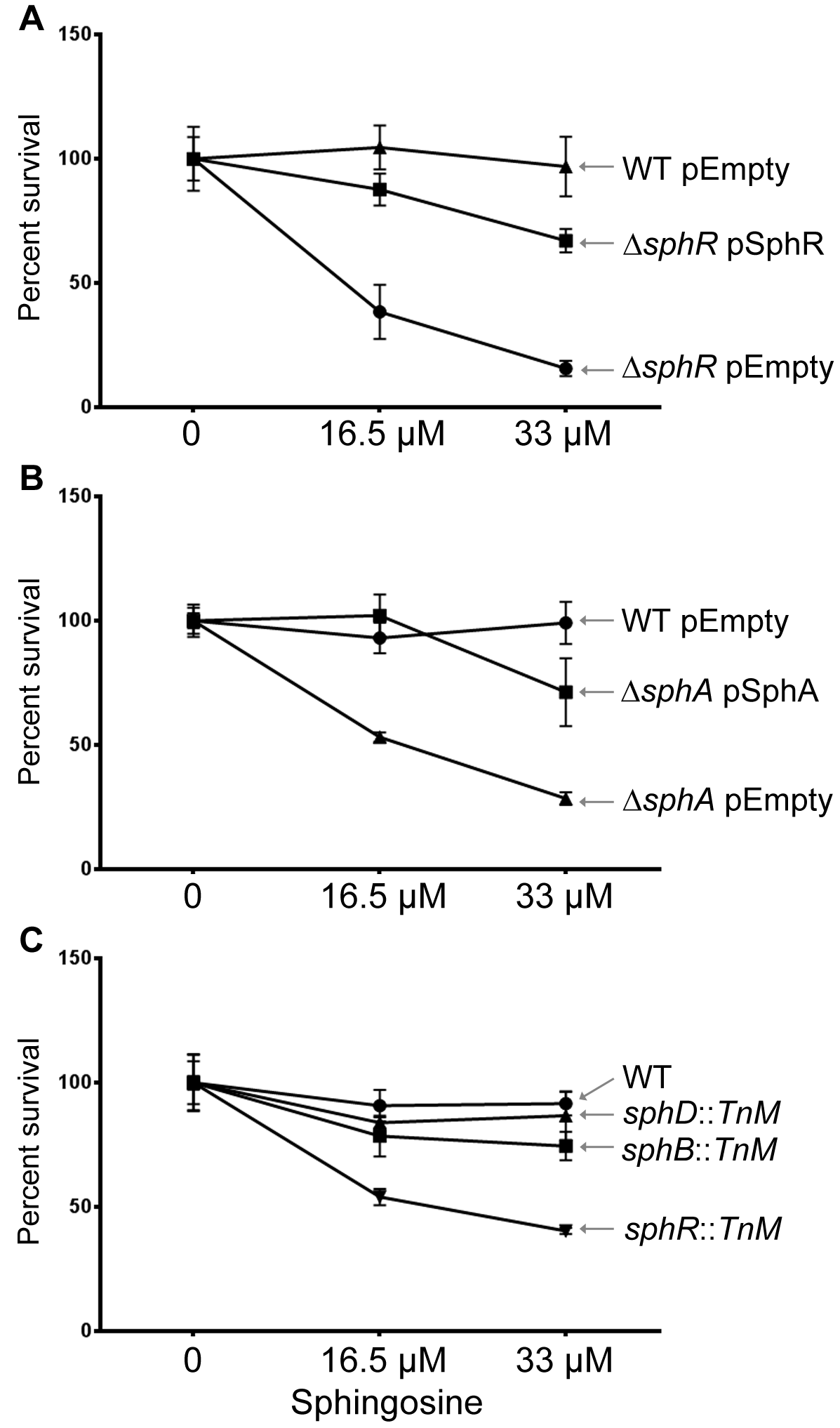

Supplement: Figure S4 — The survival of SphR-regulon mutants in sphingosine. Strains with deletions in sphA (A) and sphR (B) were susceptible to sphingosine killing and each deletion could be partially complemented with the respective gene on a plasmid compared to the empty vector (pEmpty). (C) Transposon mutants in sphB, sphD, and sphR, and a deletion mutant of cerN were compared to WT for sphingosine susceptibility. Statistical significance determined using one way ANOVA with Dunnett's post-test with wild type at each concentration being the comparator for the mutant strain data at the same concentration. p-value summaries: * for p<0.05; ** for p<0.01; *** for p<0.001; **** for p<0.0001. These experiments were performed more than three times and data shown is representative of both the scale and statistical significance levels of all experiments. (TIF) [file ppat.1003889.s004.tif]
